# IMPLEMENTATION AND VALIDATION OF STATISTICAL TESTS IN RESEARCH'S SOFTWARE HELPING DATA COLLECTION AND PROTOCOLS ANALYSIS IN SURGERY

**DOI:** 10.1590/0102-6720201600010004

**Published:** 2016

**Authors:** Carlos Henrique KURETZKI, Antônio Carlos Ligocki CAMPOS, Osvaldo MALAFAIA, Sandramara Scandelari Kusano de Paula SOARES, Sérgio Bernardo TENÓRIO, Jorge Rufino Ribas TIMI

**Affiliations:** Post-Graduation Program in Clinical Surgery, Federal University of Paraná, Curitiba, PR, Brazil

**Keywords:** Medical informatics, Statistics, Electronic protocols

## Abstract

**Background:**

The use of information technology is often applied in healthcare. With regard to scientific research, the SINPE(c) - Integrated Electronic Protocols was created as a tool to support researchers, offering clinical data standardization. By the time, SINPE(c) lacked statistical tests obtained by automatic analysis.

**Aim:**

Add to SINPE(c) features for automatic realization of the main statistical methods used in medicine .

**Methods:**

The study was divided into four topics: check the interest of users towards the implementation of the tests; search the frequency of their use in health care; carry out the implementation; and validate the results with researchers and their protocols. It was applied in a group of users of this software in their thesis in the *strict sensu* master and doctorate degrees in one postgraduate program in surgery. To assess the reliability of the statistics was compared the data obtained both automatically by SINPE(c) as manually held by a professional in statistics with experience with this type of study.

**Results:**

There was concern for the use of automatic statistical tests, with good acceptance. The chi-square, Mann-Whitney, Fisher and t-Student were considered as tests frequently used by participants in medical studies. These methods have been implemented and thereafter approved as expected.

**Conclusion:**

The incorporation of the automatic SINPE(c) Statistical Analysis was shown to be reliable and equal to the manually done, validating its use as a research tool for medical research.

## Abstract (pt)

**Racional:**

O uso de tecnologias da informação é frequentemente aplicado na área da saúde. No que diz respeito à pesquisa científica, o SINPE(c) Sistema Integrado de Protocolos Eletrônicos foi criado como ferramenta para apoiar o pesquisador facilitando a coleta padronizada e confiável. Até o momento o SINPE(c) desta não dispunha de recurso de testes estatísticos para análise automática.

**Objetivo:**

Adicionar ao SINPE(c) recursos para a realização automática dos principais métodos estatísticos utilizados na medicina.

**Métodos:**

O estudo foi dividido em quatro tópicos: verificar o interesse dos usuários para com a implementação dos testes; pesquisar a frequência de uso deles na área da saúde; realizar a implementação; e validar os resultados com os pesquisadores e seus respectivos protocolos. Foi utilizado em um grupo de usuários deste software em seus trabalhos de tese no sistema de pós-graduação estrito senso em cirurgia. Para aferir a confiabilidade dos resultados estatísticos foi utilizada comparação dos dados obtidos tanto na forma automática pelo SINPE(c) como na manual, realizada por estatístico de experiência nesse tipo de trabalho.

**Resultados:**

Houve interesse para com o uso de testes estatísticos e boa aceitação. Os testes considerados de uso frequente pelos participantes em trabalhos médicos foram qui-quadrado, Mann-Whitney, exato de Fisher e t-Student. Estes métodos foram implementados e, posteriormente, homologados conforme esperado.

**Conclusão:**

A incorporação de análise estatística automática no SINPE(c) mostrou-se confiável e de igual resultado que a estatística manual, validando seu uso como ferramenta para pesquisa na área médica.

## INTRODUCTION

After the development of the first digital computers in the 1940s, society believed they would be used as memory devices assisting in calculations and in information recovering. In the next decade, doctors and other health professionals began to realize the impact that this technology would bring to clinical practice. After six decades is remarkable the progress of computing and the contributions made to humanity, especially in the area of health^1^.

Regarding the use of scientific research focused technology there is SINPE(c) - Integrated Electronic Protocols - which purpose is support the researcher in the health field in conducting his/her search. It is a computer program designed by Dr. Osvaldo Malafaia and developed by Dr. Paul Emerson Borsato and Dr. Joseph Simon de Paula Pinto in Curitiba, PR, Brazil. This software is registered in the Brazilian National Intellectual Property Institute - INPI under number 000515432. The goal is to enable healthcare professionals develop their own electronic protocols, perform patient data collection and execute research on data collected prospectively and retrospectively. This tool also allowed the inclusion of images, videos, and complementary texts related to data collect.^3^

After exploratory research with SINPE(c) users, it was found that the analytical statistics feature would provide benefits to researchers allowing the use of statistical tests directly by the researchers.

The interest in the use of data in health research and evaluation has increased with the growth of availability of large databases and computer programs that enable its integrated use^4^. The statistical analysis of the results obtained in a particular study is a very important tool to validate such data^5^.

From the identified need was sought to get through research reviewed in the health area, the statistical tests most commonly used for later deployment on SINPE(c). The use of statistics in health care work is common. This application provides accurately, evidence of statistical hypothesis and conclusion numerically proven facts.

The objective of this study was to provide easier electronic way by means of specific developed software to collect and realize data analysis, improving results by using statistical tests normally related to health care.

## METHOD

After survey among SINPE(c) users about the interest of using statistical tests within the software, and having yes as answer, it was searched in the literature which were the statistical tests most commonly used in health care.

At the beginning of February 2011, search was done in the published issues of Brazilian Archives of Digestive Surgery journal in order to identify the methods used in scientific papers. Original articles of 2009 and 2010 were selected from this journal and were consolidated in Microsoft Office Excel(r) spreadsheet, disregarding the cases where it was applied variance analysis. The tab and lifting of the results was completed at the end of that month. For implementation of statistical methods, literature references were used in order to identify and analyze the formulas and its full operation.

Worksheets in Microsoft Office Excel(r) containing the formulas and examples from literature were created for each statistical method to support the development. 

After the creation of this material was held the comparison of results obtained in the spreadsheet with what appeared in the literature; this way, problems of logic and simulations were performed. Later, there was the implementation of the chi-square test, Student's t, Fisher exact test and Mann-Whitney. Before to implementation in SINPE(c) Analyzer tool was decided transcribe their formulas in Microsoft Office Excel(r) to provide testing before the final development. After the sheet elaboration the result obtained from the literature was confronted and was confirmed in both cases. After this phase, the test was implemented in SINPE(c) Analyzer module. This implementation started and was completed in January 2012 and passed by correction in August 2013.

The tests were performed with the Multidisciplinary Protocol for Bariatric Surgery of PhD Denise Bopp Nassif; at this moment, this researcher was not involved, because the purpose of the tests was to confront if the calculations were consistent with the implemented. To execute the validation of calculations, there was a selection of two items Multidisciplinary Protocol for Bariatric Surgery; the data was tabulated in the backing sheet with the Mann-Whitney test calculations and after this the same test was executed using functionality developed in SINPE(c) Analyzer. There was equality in the comparison of results.

## RESULTS

Between 2009 and 2010 were analyzed 57 original articles of the journal. In this analysis were obtained 16 statistical methods and eight of them were used only once. During 2009, 12 articles had no statistical analysis or did not identify the statistical method used. The same number was repeated for 2010.

The four main tests used were: t-Student with 10 occurrences; Fisher with 10 occurrences; Chi-square with eight events and Mann-Whitney with eight occurrences. The t -Student and Fisher tests were used in 17.5% and the methods chi-square and Mann- Whitney in 14% at the papers during the referred years.

The chi-square test was validated by Rodrigo Hamerschmidt in his Master's degree thesis in which tests manually done by statistical professional's services confirmed the result obtained through the software which χ^2^=7.93 and p=0.005.

The validation process of the Mann-Whitney test was supported by Dr. Marina Serrato Coelho Fagundes in her electronic protocol called Multi-professional Protocol Disease in the field of otolaryngology specific in Rhinoplasty. The test was not selected by the statistic's professional; the validation was done confronting the manual result with the one provided by the software, getting the same results U_1_=253.5 and U_2_=70.5. Considering the results comparison was possible to validate the test.

The validation process of Fisher test was done considering the services of statistical professionals at medical protocol from the Dr. Luiz Alberto Zago Filho with the protocol Invasion of Neovessels for Vitreous Outcome. The result was p=0.18467 for the calculation from statistic's professional and also for the software. These results were validated and presented in the doctoral thesis of the researcher.

The t-Student test was supported by the services of professional statistic medical researcher Mrs. Marina Serrato Coelho Fagundes for the rhinoplasty protocol analysis of nasal projection. In all calculations made ​​by statisticians was obtained the value of t=0.82 and p=0.421. After running the software the results were identical. In this way the results of the professional statistical and also the results implemented t-Student in SINPE(c) Analyzer module were confirmed.

## DISCUSSION

The "gap" between statistics and clinical health professionals in relationship to mathematics, understanding, test's comprehension and language used by statisticians during the communication with them, can justify the disinterest for a considerable part of researchers and students about statistic^5^.

The health field is one of the largest in most countries and can significantly benefit from high-quality data in real time independent of location. However, many health professionals are not familiar with the solutions of Information Technology, business and information or are reluctant to apply them in their ​​work area^6^.

Biostatistics is the application of statistics to the understanding of health and biology; it provides powerful tools for the development of research questions, design studies, refining the measurements, data analysis and interpretation of results^7^.

From the 40s, the statistical research turns to solve problems involving various aspects of inference, each having its application in specific situations^8^. The use of information technology enables to record large volume of information, taking the advantage of speed and reliability of their calculations.

Every sample from surveys should be analyzed with statistical software algorithms for complex data analysis^9^.

The computer programs have also been used in nutrition research, statistical analysis, data reporting, nutrients and dietary evolution analysis programs. In addition, researchers use computer programs for data protocol^10^. The creation of electronic databases in research centers, from electronic protocols, allows large-capacity storage and information processing, as well as facilitating the access and retrieval data and allows realization of prospective papers of high quality^11^.

For independent confirmation and validation, was sought to compare statistic results obtained manually by related professionals with the ones offered by SINPE(c) software using the same statistical tests, but automatically. Researchers only forwarded their data indicating the tests to be used in order to validate, through the confrontation, the automatic and manual results.

The creation of a guide to facilitate the researcher in choosing by himself the statistical method is the suggestion for future study, as well as the addition of other tests to amplify SINPE(c) statistic spectrum. 

## CONCLUSION

It was provided to health care professionals electronic data collection using validated specific software to improve analysis with automatic and computerized use of statistical tests.
